# In vitro characterization of PrismaLung+: a novel ECCO_2_R device

**DOI:** 10.1186/s40635-020-00301-7

**Published:** 2020-05-13

**Authors:** Ingeborg Hospach, Jacques Goldstein, Kai Harenski, John G. Laffey, Dominique Pouchoulin, Manuela Raible, Stefanie Votteler, Markus Storr

**Affiliations:** 1 Baxter International, Research and Development, Holger-Crafoord-Str. 26, 72379 Hechingen, Germany; 2 Baxter World Trade SPRL, Acute Therapies Global, Braine-l’Alleud, Belgium; 3 Baxter, Baxter Deutschland GmbH, Unterschleissheim, Germany; 4 Anaesthesia and Intensive Care Medicine, School of Medicine, NUI Galway, Galway, Ireland; 5 Baxter, Gambro Industries, Meyzieu, France

**Keywords:** Acute respiratory distress syndrome (ARDS), Chronic obstructive pulmonary disease (COPD), CO_2_ removal, Extracorporeal CO_2_ removal (ECCO_2_R), Gas exchange, Hypercapnic respiratory failure, Lung protective ventilation, Mechanical ventilation, Tidal volume

## Abstract

**Background:**

Invasive mechanical ventilation is lifesaving in the setting of severe acute respiratory failure but can cause ventilation-induced lung injury. Advances in extracorporeal CO_2_ removal (ECCO_2_R) technologies may facilitate more protective lung ventilation in acute respiratory distress syndrome, and enable earlier weaning and/or avoid invasive mechanical ventilation entirely in chronic obstructive pulmonary disease exacerbations. We evaluated the in vitro CO_2_ removal capacity of the novel PrismaLung+ ECCO_2_R device compared with two existing gas exchangers.

**Methods:**

The in vitro CO_2_ removal capacity of the PrismaLung+ (surface area 0.8 m^2^, Baxter) was compared with the PrismaLung (surface area 0.35 m^2^, Baxter) and A.L.ONE (surface area 1.35 m^2^, Eurosets) devices, using a closed-loop bovine blood–perfused extracorporeal circuit. The efficacy of each device was measured at varying pCO_2_ inlet (p_in_CO_2_) levels (45, 60, and 80 mmHg) and blood flow rates (*Q*_B_) of 200–450 mL/min; the PrismaLung+ and A.L.ONE devices were also tested at a *Q*_B_ of 600 mL/min. The amount of CO_2_ removed by each device was assessed by measurement of the CO_2_ infused to maintain circuit equilibrium (CO_2_ infusion method) and compared with measured CO_2_ concentrations in the inlet and outlet of the CO_2_ removal device (blood gas analysis method).

**Results:**

The PrismaLung+ device performed similarly to the A.L.ONE device, with both devices demonstrating CO_2_ removal rates ~ 50% greater than the PrismaLung device. CO_2_ removal rates were 73 ± 4.0, 44 ± 2.5, and 72 ± 1.9 mL/min, for PrismaLung+, PrismaLung, and A.L.ONE, respectively, at *Q*_B_ 300 mL/min and p_in_CO_2_ 45 mmHg. A Bland–Altman plot demonstrated that the CO_2_ infusion method was comparable to the blood gas analysis method for calculating CO_2_ removal. The resistance to blood flow across the test device, as measured by pressure drop, varied as a function of blood flow rate, and was greatest for PrismaLung and lowest for the A.L.ONE device.

**Conclusions:**

The newly developed PrismaLung+ performed more effectively than PrismaLung, with performance of CO_2_ removal comparable to A.L.ONE at the flow rates tested, despite the smaller membrane surface area of PrismaLung+ versus A.L.ONE. Clinical testing of PrismaLung+ is warranted to further characterize its performance.

## Background

Patients with severe acute hypoxemic and/or hypercapnic respiratory failure require invasive mechanical ventilation (IMV) to facilitate gas exchange and to support breathing. While IMV may be lifesaving in this setting, it is associated with significant short- and long-term side effects. Consequently, there is considerable interest in developing strategies such as extracorporeal CO_2_ removal (ECCO_2_R), which can facilitate CO_2_ removal [1], or extracorporeal membrane oxygenation (ECMO), which, in addition, provides oxygenation in instances of severe hypoxemic respiratory failure [2]. These approaches may enable reductions in the intensity and/or the duration of IMV in these patients.

In patients with severe hypoxemia, particularly those with acute respiratory distress syndrome (ARDS), the loss of alveolar ventilation capacity due to alveolar consolidation, edema and/or collapse contributes to the need for ventilatory support [3]. The discovery that high tidal and minute ventilation strategies can cause harm—termed “ventilator-induced lung injury” (VILI )[4–6]—has led to the use of lung “protective” ventilation (LPV) strategies, where low tidal volumes (4–8 mL/kg of per body weight [PBW ][7] versus 10–15 mL/kg of PBW in conventional mechanical ventilation [MV ][6]) decrease lung stretch, reduce VILI [8], and can potentially improve survival and reduce mortality in patients with acute lung injury and ARDS [6, 9]. Amato et al. showed that lower driving pressure was the physical variable that best correlated with survival in patients with ARDS [10]; higher positive end-expiratory pressure (PEEP), lower peak and plateau pressures, and lower respiratory rate, may also be associated with improved survival [11, 12].

The use of lower tidal and minute volumes with LPV strategies is limited by the resultant respiratory acidosis [13–15]. The rationale to integrate ECCO_2_R into the management of severe ARDS is to allow more protective ventilation, i.e., providing very low tidal volumes (*V*_T_) (less than 6 mL/kg PBW) with conventional MV, while avoiding extreme levels of respiratory acidosis. Arterial CO_2_ tensions are generally maintained in the range 45–60 mmHg rather than targeting normocapnia with this approach [16]. The potential for use of ECCO_2_R in patients with ARDS has been evidenced in a number of clinical studies [17–19], indicating it may be an effective strategy in ARDS management and a viable option to further reduce tidal and minute volumes in these patients [15, 16].

In patients with acute exacerbations of chronic obstructive pulmonary disease (aeCOPD), where hypercapnia is predominant, non-invasive positive pressure ventilation (NIV) is used as a first-line strategy in order to avoid MV [20]. Use of NIV has been reported to reduce mortality by approximately 70% [21]; however, in some patients, additional assistance is required to prevent the need for intubation [22]. NIV fails in almost 40% of cases, and patients must undergo endotracheal intubation and IMV to restore adequate gas exchange [22–25]. There is increasing clinical evidence supporting the use of low-flow, partial ECCO_2_R for patients experiencing aeCOPD who are failing support with NIV [22], avoiding the need for IMV and/or decreasing the length of time on the ventilator [26].

Advances in extracorporeal device technologies have made selective ECCO_2_R devices a less invasive and more feasible option than ECMO, with several devices clinically available that utilize blood flow rates between 180 mL/min and 1700 mL/min [27]. However, these devices were historically designed for use as oxygenators for ECMO treatment in the neonatal or pediatric setting, rather than being optimized for CO_2_ removal [28]. Here, we describe a newly developed ECCO_2_R device, the PrismaLung+ (Additional file 1: Figure S1), created specifically for CO_2_ removal. We compared the in vitro CO_2_ removal rates during low blood flow (*Q*_B_ 200–450 mL/min) of three devices: PrismaLung+ (Baxter), PrismaLung (Baxter), and Eurosets A.L.ONE (Eurosets), and during a *Q*_B_ of 600 mL/min for the PrismaLung+ and A.L.ONE devices [29–31]. We hypothesized that PrismaLung+ with a membrane surface area of 0.8 m^2^ provides significantly higher CO_2_ removal rates than PrismaLung (surface area 0.35 m^2^), whereas we expected similar performance for PrismaLung+ and the A.L.ONE device (surface area 1.35 m^2^), since with increasing membrane surface area, the low blood flow rates limit CO_2_ removal.

## Methods

### Experimental set-up

In vitro experimentation to determine CO_2_ removal rates was performed using three different ECCO_2_R devices: PrismaLung+ (Baxter), PrismaLung (Baxter), and Eurosets A.L.ONE (Eurosets) (Table 1). The devices were selected as they had the same membrane composition, i.e., polymethylpentene hollow-fiber mats, in order to remove this potential source of variability from the experiments. Five test devices of each type were investigated. The total surface areas of the gas exchange membranes are PrismaLung+0.8 m^2^, PrismaLung, 0.35m^2,^ and A.L.ONE, 1.35 m^2^.

**Table 1:**
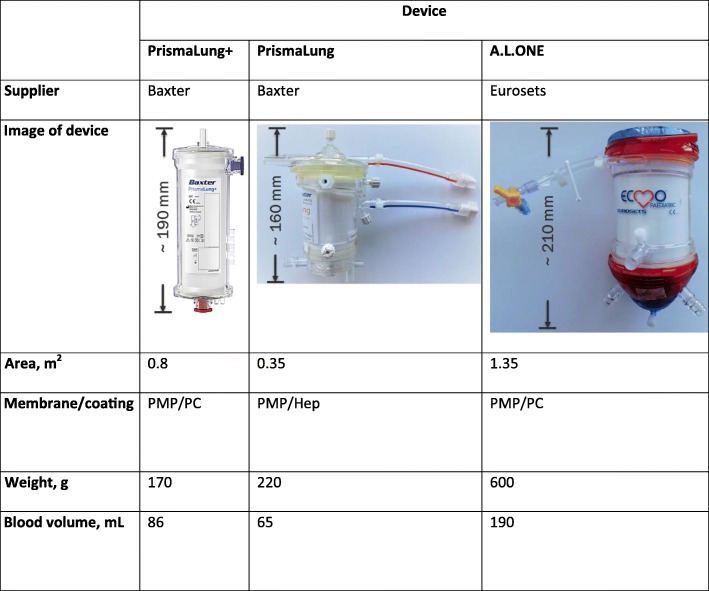
Characteristics of the different test gas exchangers

*PMP* polymethylpentene hollow-fiber mats, gas-permeable membrane, *PC* phosphorylcholin, *Hep* heparin

**Fig. 1:**
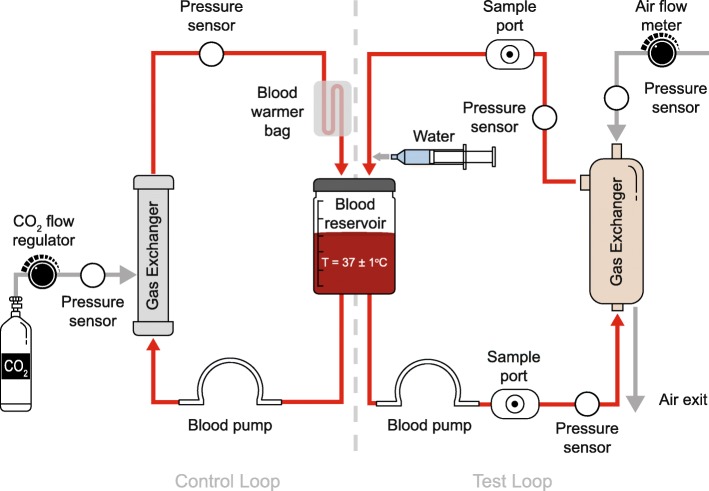
In vitro test setup for sample taking to determine CO_2_ removal performance

Test media bovine blood parameters were adjusted as listed in Table 2. NaCl and NaHCO_3_ solutions were used to adjust the required ranges of blood parameters. The experimental setup was a closed-loop circuit in which a continuous CO_2_ infusion balanced the CO_2_ removal from the test gas exchanger to establish a steady-state condition and allowing the CO_2_ removal rate to be determined (Fig. 1). The total amount of blood used in the circuit was approximately 600–700 mL. The test setup comprised the following: blood reservoir, 250 ml Duran glass bottle (Schott AG, Germany) with a temperature sensor Pt100 (Technetics, Germany); tubing (Promedt, Germany) with inserted septum as sample port and valves; 2 × peristaltic blood pumps (made in-house, Baxter, Germany); datalogger for sensor read-out mikromec® logger (Technetics, Germany); control loop: gas exchanger for CO_2_ input, PrismaLung (Baxter, Germany) (closed at gas outlet with plugs), a Thermax blood warmer bag (Baxter, France) inside an in-house made holder, and water bath with thermostat EH (Julabo, Germany); CO_2_ gas bottle ≥ 99.5% purity (Linde, Germany) including pressure regulator and gas tubing; CO_2_ mass flow regulator GSC-A9TA-BB22 (Vögtlin, Switzerland); 2 × pressure sensor, PE2 bar (Technetics, Germany); Test loop: sweep gas mass flow regulator GSC-C9TA-BB12 (Vögtlin, Switzerland); 3 × pressure sensor, PE1 bar (Technetics, Germany); syringe pump 540270 (TSE Systems, Germany); compressed air as sweep gas (in-house) including pressure regulator and tubing. Blood samples were analyzed with an ABL 90 blood gas analyzer (Radiometer, Germany).

**Table 2:** Defined test conditions for each test gas exchanger

A. Test media bovine blood	
	From abattoir, stored at 4 °C • Anticoagulation: Heparin (~ 5 U/mL) • Filtered (50 μm, polyamine, SEFAR-NITEX) • Total Proteins: 60 ± 5 g/L • Hct: 31–33% • Viscosity, blood plasma: 1.50–1.64 mm^2^/s • c(Base)B: 0 ± 5 mmol/L
B. Set-up	
Blood flow rates (*Q*_B_)	200, 300, 450 mL/min ± 4.3%*
p_in_CO_2__ref	45, 60, 80 mmHg ±10%
Sweep gas flow rates	10 ± 1 L/min
Temperature in blood reservoir	37 ± 1 °C
ctHb	12 ± 1 g/dL
c(Base) B	0 ± 5 mmol/L
Na^+^	Initial concentration ± 5 mmol/L

A. Adjusted parameters of test media before experimentation. B. Set and controlled parameters during experimentation

*c(Base) B* base excess in blood, *ctHb* total hemoglobin concentration, *Hct* hematocrit, *Na*^*+*^ sodium ion, *pCO*_*2*_ partial pressure of carbon dioxide, *p*_*in*_*CO*_*2*_*_ref* target inlet pCO_2_, *Q*_*B*_ blood flow rate

*The PrismaLung+ and A.L.ONE devices were additionally tested at *Q*_B_ 600 mL/min and p_in_CO_2_ 45 mmHg

Before use, an integrity test of the CO_2_ infusion circuit was performed, after which the whole test setup, including test devices, was primed with saline or dialysate solution (e.g., Prismasol 2, Baxter) to remove all air. The setup was then filled with bovine blood and all saline or dialysate solution was replaced. The test circuit comprised a central reservoir filled with 200–300 mL of blood as well as two loops. The control loop had a gas exchanger connected to a CO_2_ supply, which was used to achieve the targeted p_in_CO_2_ levels. In addition, a blood warming system, where the blood warmer bag was submerged in a water bath, was used to maintain blood pool temperature at 37 ± 1 °C. The control loop was fed through the central reservoir then connected to the test loop in which the test device was attached, and CO_2_ removal was determined. Loss of water due to evaporation through the membrane and into the sweep gas was balanced by infusion of reverse osmosis water. The sodium concentration was kept constant throughout experimentation, as analyzed by the blood gas analyzer, to maintain a constant water flow.

### Measurements/data collection

When the test was initiated, the blood flow of the control loop was set to 500 mL/min and the sweep gas flow was set at the targeted rate. The CO_2_ inlet flow was adjusted in a stepwise fashion to maintain p_in_CO_2_ at the targeted value and to reach steady-state conditions (constant values for p_in_CO_2_, CO_2_ inflow rate, CO_2_ removal rate). The pCO_2_ value was measured by blood gas analysis, after samples were taken at the blood inlet of the test device. Following an equilibration time of at least 13 minutes, during which CO_2_ removal from the test circuit was demonstrated to be balanced by CO_2_ addition to the control loop, CO_2_ removal rate was determined based on the CO_2_ inflow rate. If blood samples were taken, a syringe with a volume of 0.5–1 mL was used. On average, no more than 2–3 blood sample measurements were necessary to confirm a steady state, which is below 0.5% of the total circuit blood volume.

For each test device, at all requested test parameters (9 settings of varying *Q*_B_ and p_in_CO_2_, Table 3 in the Appendix), measurements were taken at the inlet and outlet, with samples taken in triplicate. PO_2_ inlet values (160–183 mmHg) indicated that the blood used in this study was oxygen saturated. All devices were tested at *Q*_B_ 200, 300, and 450 mL/min, with additional testing of the PrismaLung+ and A.L.ONE devices at *Q*_B_ 600 mL/min and p_in_CO_2_ 45 mmHg. Test conditions are outlined in Table 2.

The primary method utilized for measuring CO_2_ removal was the infusion method, which was validated using the blood gas analysis method. In the infusion method, normalized CO_2_ removal rate (JCO_2(inf)_) was determined based on the CO_2_ input flow rate at equilibrium, controlled by sample taking and analysis at the inlet. The blood gas analysis method utilized the same setup as the infusion method, but blood samples were taken additionally after the test device, at the outlet. Only samples, where measured pCO_2_ and/or pH were inside the reportable range (i.e., pCO_2_ > 12 mmHg, pH < 7.85) were used to validate the data from the infusion method.

Prior to the series of experiments, the suitability of the analyzer, with respect to its intended use, was verified through the measurement principles of the blood gas analyzer [32, 33], to ensure that the device was able to measure bovine parameters. Furthermore, the same device was used for all replicates. Measurements taken included the analysis of ctHb (g/dL), pH, pCO_2_ (mmHg), pO_2_ (mmHg), and FMetHb (%) (Table 3 in the Appendix). Following experimentation, an integrity test of the circuit was again performed to confirm its CO_2_ gas integrity.

### Data analysis

Mean values were determined from triplicate measurements.

In the infusion method, JCO_2(inf)_ was determined based on CO_2_ input flow rate, using the following equation:

$$ {\mathrm{JCO}}_{2\left(\operatorname{inf}\right)}\left(\mathrm{mL}/\min \right)={\mathrm{QCO}}_2\times \left({\mathrm{p}}_{\mathrm{in}}{\mathrm{CO}}_{2\left(\mathrm{ref}\right)}/{\mathrm{p}}_{\mathrm{in}}{\mathrm{CO}}_{2\left(\mathrm{inlet}\right)}\right) $$

where QCO_2_ is the CO_2_ input flow within the control loop, p_in_CO_2(ref)_ is the target inlet pCO_2_ of 45, 60, or 80 mmHg, and p_in_CO_2(inlet)_ is the actual partial pressure of CO_2_ in the blood reservoir or gas exchanger inlet. p_in_CO_2_ was normalized to reduce variability in measurements resulting from small deviations from target p_in_CO_2_ values (± 10%).

QCO_2_ values are referred to in mL/min under normal conditions (0 °C, 1013 mbar) and are re-calculated where appropriate, applying the ideal gas equation:

$$ Q(T)={\mathrm{QCO}}_2\times \left(273.15+T\right)/273.15 $$

where *Q* (*T*) is the volumetric flow at a defined temperature (*T*, in °C). The temperatures used for calculation ranged from 0 to 37 °C. Note: the atmospheric pressure is assumed to be constant at 1013 mbar.

In addition, using the more commonly utilized blood gas analysis method, the normalized CO_2_ removal rate (JCO_2(BGA)_) was determined according to the following equation:

$$ {\mathrm{JCO}}_{2\left(\mathrm{BGA}\right)}\left(\mathrm{mL}/\min \right)=\left({V}_{\mathrm{m}}\times {Q}_{\mathrm{B}}\times \Big[{\mathrm{ctCO}}_{2\left(\mathrm{inlet}\right)}-{\mathrm{ctCO}}_{2\left(\mathrm{outlet}\right)}\right]\Big)\times \left({\mathrm{p}}_{\mathrm{in}}{\mathrm{CO}}_{2\left(\mathrm{ref}\right)}/{\mathrm{p}}_{\mathrm{in}}{\mathrm{CO}}_{2\left(\mathrm{inlet}\right)}\right) $$

where *V*_m_ is the temperature-dependent molar volume; *Q*_B_ is the blood flow within the test loop; ctCO_2_ is the total blood concentration of CO_2_ (given by the blood gas analyzer, derived from pH, pCO_2_, saturation of oxygen sO_2_, and hemoglobin concentration); p_in_CO_2(ref)_ is the target inlet pCO_2_ of 45, 60, or 80 mmHg; and p_in_CO_2(inlet)_ is the partial pressure of CO_2_ in blood.

CO_2_ removal rates were additionally calculated in units of mmol/min to remove any dependency of reported values upon pressure and reference temperature.

### Statistical analysis

A total of 5 test runs were performed for each device and parameter settings. Data are expressed as mean ± SD and the normal distribution of the data sets was assessed using the Kolmogrov–Smirnov Test (α = 0.05). CO_2_ removal performance results were compared using an ANOVA test with *p* values of < 0.05 considered as indicating a significant difference. Bland–Altman analysis was used to compare the two different performance test methods, generated using Sigmaplot software [34, 35]. A linear regression comparing both methods was additionally used. A paired *t* test was used to compare data obtained via the infusion and blood gas analysis methods.

## Results

### CO_2_ removal rates

#### Performance across the different ECCO_2_R devices

**Fig. 2:**
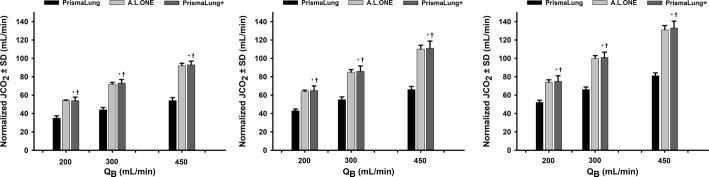
CO_2_ removal performance as a function of p_in_CO_2_ and *Q*_B_. Assessed using the infusion method for the three devices at 37 °C, across the blood flow rates (*Q*_B_) 200, 300, and 450 mL/min at p_in_CO_2_ levels of **a** 45 mmHg, **b** 60 mmHg, and **c** 80 mmHg. p_in_CO_2_, partial pressure of carbon dioxide at the inlet; *Q*_B_, blood flow rate. Data are plotted as mean values ± SD. *p > 0.05 PrismaLung+ vs. A.L.ONE; †*p* < 0.05 PrismaLung+ vs. PrismaLung

The CO_2_ removal rates of the different devices were analyzed using the infusion method at p_in_CO_2_ levels (45, 60, and 80 mmHg) and *Q*_B_ (200, 300, and 450 mL/min), at 37 °C (Fig. 2 a–c, Table 4 in the Appendix). The A.L.ONE and PrismaLung+ devices provided comparable CO_2_ removal rates across the range of different test conditions (*p* > 0.05, not significant). For both devices, removal rates were significantly higher than those observed with the PrismaLung device (*p* < 0.05).

CO_2_ removal rates at an increased blood flow rate of 600 mL/min were additionally evaluated for the PrismaLung+ and A.L.ONE devices only and were comparable for both devices (*p* > 0.05) (Additional file 2: Figure S2). At a p_in_CO_2_ of 45 mmHg, at 37 °C, the mean CO_2_ removal rates at a blood flow rate of 600 mL/min were 106 ± 3.8 mL/min and 106 ± 5.8 mL/min for the PrismaLung+ and A.L.ONE devices, respectively.

**Fig. 3:**
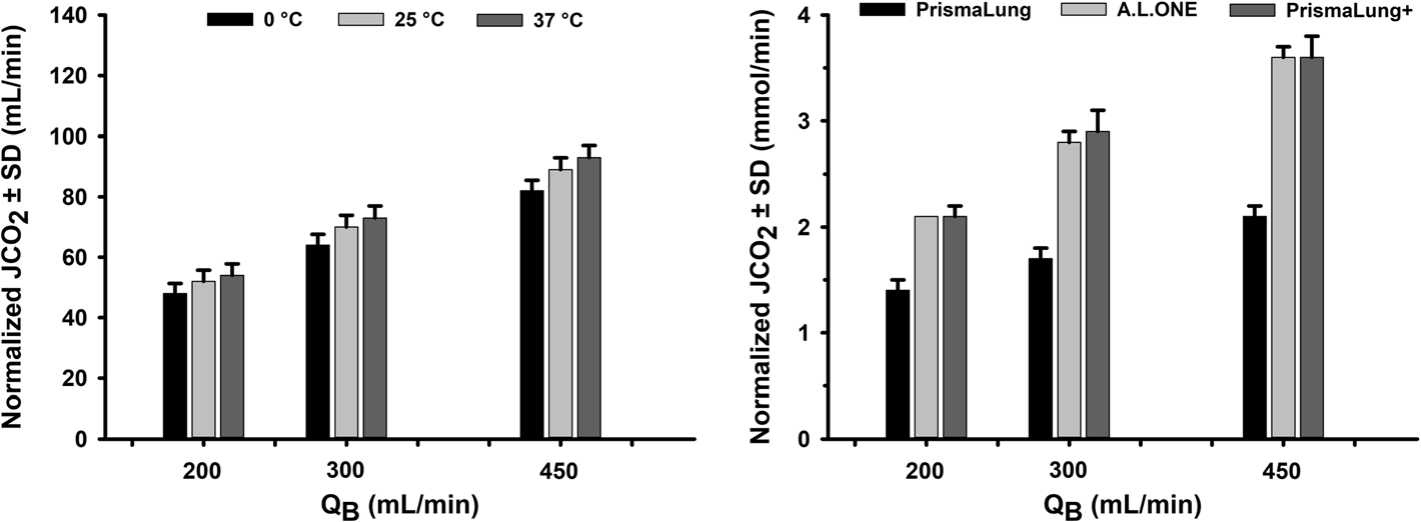
CO_2_ removal performance as a function of temperature. Assessed using the infusion method for **a** PrismaLung+ at a p_in_CO_2_ of 45 mmHg and *Q*_B_ 200–450 mL/min, referenced to 0, 25, or 37 °C, and **b** for all tested gas exchangers at 37 °C, at a p_in_CO_2_ of 45 mmHg, across *Q*_B_ 200–450 mL/min, in units of mmol/min. p_in_CO_2_, partial pressure of carbon dioxide at the inlet; *Q*_B_, blood flow rate. Data are plotted as mean values ± SD

As the volume flow of gases, i.e., the CO_2_ removal rate, is temperature- and pressure-dependent, data were calculated at standard reference conditions, 0 °C and 25 °C (STP as defined by IUPAC), in addition to the physiological conditions, 37 °C, for the PrismaLung+ device at a p_in_CO2 of 45 mmHg and *Q*_B_ range of 200–450 mL/min (Fig. 3a). Results illustrate the dependence of CO_2_ removal on the chosen reference temperature. By definition, calculation of CO_2_ removal rates in mmol/min across *Q*_B_ 200–450 mL/min, at a p_in_CO_2_ of 45 mmHg, is independent from any reference temperature (Fig. 3b).

### Pressure drop levels

**Fig. 4:**
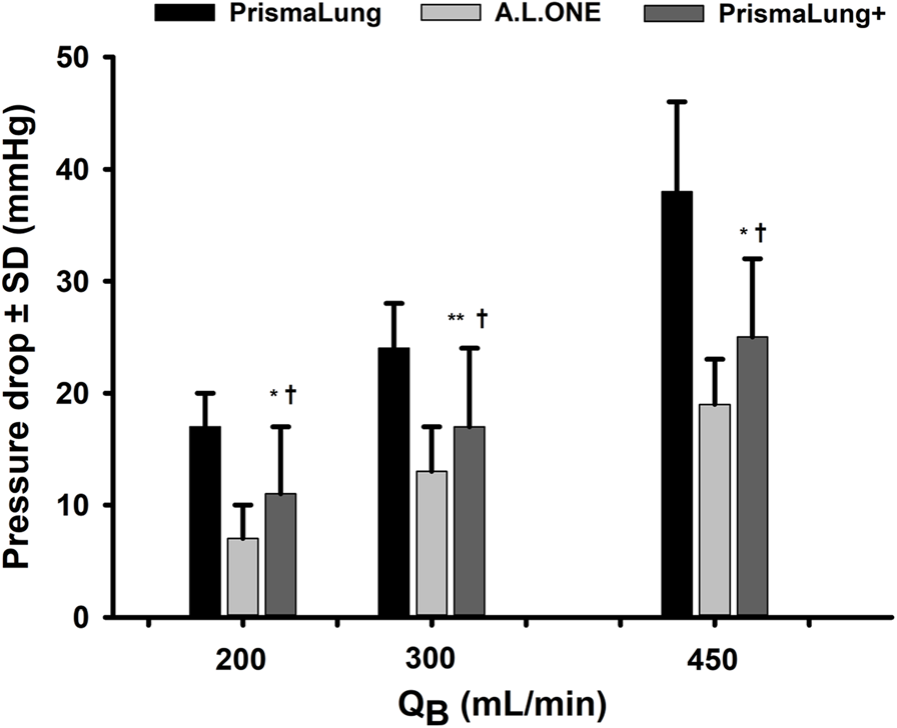
Pressure drop blood side across the devices. *Q*_B_, blood flow rate. Data are plotted as mean values ± SD. *p < 0.05; **p > 0.05 PrismaLung+ vs. A.L.ONE; †*p* < 0.05 PrismaLung+ vs. PrismaLung

To examine the blood flow resistance for each device, pressure drop was analyzed at the blood side, for all p_in_CO_2_ levels (45, 60, and 80 mmHg). Pressure drop was observed to be largest for the PrismaLung, and lowest for the A.L.ONE device, being 17 (±3), 24 (±4), and 38 (±8) mmHg (±SD) for PrismaLung; 11 (±6), 17 (±7), and 25 (±7) mmHg (±SD) for PrismaLung+; and 7 (±3), 13 (±4), and 19 (±4) mmHg (±SD) for the A.L.ONE device, at blood flow rates of 200, 300, and 450 mL/min, respectively (Fig. 4). All comparisons for the PrismaLung+ versus PrismaLung, and PrismaLung+ versus A.L.ONE devices, were significantly different (*p* < 0.05), except for the PrismaLung+ versus A.L.ONE devices at a *Q*_B_ of 300 mL/min (*p* > 0.05). The differences seen across the devices are likely driven by the variances in the surface areas, with PrismaLung having the smallest surface area (0.35 m^2^), followed by PrismaLung+ (0.8 m^2^) and then A.L.ONE (1.35 m^2^).

### Analysis using the infusion and blood gas methods

**Fig. 5:**
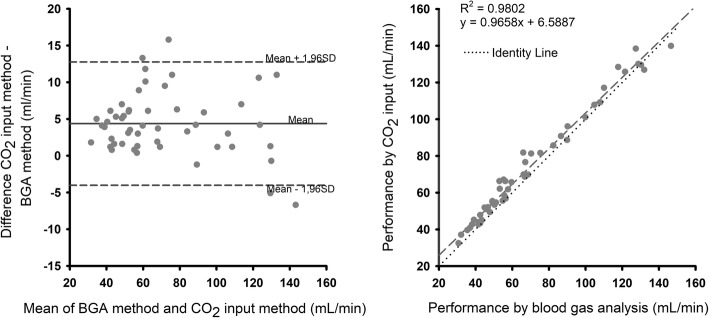
Comparison of CO_2_ removal performance using the infusion and blood gas analysis methods. Assessed across all tested devices and parameter settings at 37 °C. **a** Bland–Altman analysis to demonstrate the relationship between the BGA method and the CO_2_ input method. Mean = 4.2 mL/min, SD = 4.4 mL/min, Limits of Agreement (95%) = − 4.4 mL/min, 12.9 mL/min. **b** Linear regression analysis comparing the two test methods: BGA method and CO_2_ input method. Slope not significantly different from 1.0. BGA, blood gas analysis; SD, standard deviation

The CO_2_ removal performances were analyzed using the infusion and blood gas analysis methods. However, at the lower blood flow rates, many of the outlet samples were below the measuring range of pCO_2_, among others, using the blood gas analyzer method. A comparison of the two methods using a Bland–Altman analysis (Fig. 5a) and linear regression analysis (Fig. 5b) across the different p_in_CO_2_ levels (45, 60, and 80 mmHg) and blood flow rates (200, 300, and 450 mL/min), using valid data within the reportable range of the blood gas analyzer used, indicated a linear relationship between the data obtained by the two methods, suggesting comparability. Statistical analysis revealed that CO_2_ removal performance values obtained with the infusion method were, on average, 4.2 mL greater than the values obtained with blood gas analysis (*p* < 0.05). In addition, the difference was shown to be independent of the test conditions and a constant offset between the two methods. The infusion method was used for the analysis of the full data set as the two methods are similar in terms of validity.

## Discussion

In this in vitro study, the CO_2_ removal performance of the new PrismaLung+ device was comparable to the A.L.ONE device, with both devices demonstrating CO_2_ removal rates ~ 50% greater than the PrismaLung device. The performance of the three devices was consistent over a range of blood p_in_CO_2_ levels and at flow rates from 200 to 450 mL/min, with both the PrismaLung+ and A.L.ONE devices also performing comparably at the higher flow rate of 600 mL/min. CO_2_ removal data obtained with the CO_2_ infusion method were comparable to those obtained with the blood gas analysis method. The resistance to blood flow across the test device, as measured by pressure drop, varied as a function of blood flow rate, being greatest for the PrismaLung, intermediate for the PrismaLung+, and lowest for the A.L.ONE device, most likely driven by the differences in the surface areas. Taken together with prior clinical studies of ECCO_2_R devices [17–19, 22], these findings suggest that the PrismaLung+ may be an effective device and further testing in the clinical setting is warranted.

### Rationale for CO_2_ removal

ECCO_2_R technologies may have important roles in the management of patients with ARDS and patients with aeCOPD. ECCO_2_R can help facilitate lung-protective strategies by enabling very low *V*_T_ (< 6 mL/kg PBW) ventilation [36, 37]. The safety and feasibility of ECCO_2_R has been demonstrated in multiple studies [16, 18, 19] of patients with ARDS, with reduced lung injury and benefits in terms of pulmonary inflammation with low *V*_T_ ventilation [17]. Several studies also support the use of ECCO_2_R in patients with aeCOPD requiring ventilatory support [38–42].

### Lower flow ECCO_2_R devices

Devices with reduced blood flow requirements will, by design, be less efficient at removing CO_2_ than higher flow devices, but they do have several advantages. The PrismaLung+ device in this study has a design tailored to specifically remove CO_2_. The lack of a need for a heat exchanger inside the device allows for reduced size and weight, given that a heat exchanger is available for use next to the machine during treatment. The new PrismaLung+ device has the lowest ratio of blood volume to membrane surface of the tested devices reducing the risks associated with large extracorporeal blood volumes. The removal of the heater also allows for a streamlined design, which should reduce the potential for pooling and low flows of blood within the device. Further aspects of the device design, including the fluid path and dimensional parameters, have been developed to enable an intended operating blood flow of 200 to 450 mL/min. Namely blood flow velocity distribution was calculated to avoid stagnant areas or areas with very low blood flow velocity and to ensure that channeling of the blood did not occur. The residual volume space is smaller than other devices, minimizing the space for blood to clot. The ratio of CO_2_ removal rate to blood volume of the PrismaLung+ device allows for optimized performance at these flow rates. It has been shown that an extracorporeal CO_2_ removal rate of 51 ± 26 mL/min was associated with an increase in PaCO_2_ from 43 ± 8 to 53 ± 9 mmHg when applying low tidal volume ventilation (*V*_T_ = 4 mL/kg) in patients with mild-to-moderate ARDS [43]. Whereas a mean CO_2_ removal of 81 ± 9 mL/min enabled a reduction in *V*_T_ to 4.29 ± 9 mL/min without an increase in PaCO_2_ of more than 10% [19]. Therefore, larger CO_2_ removal rates are desirable to allow ultraprotective ventilation in ARDS patients without a significant increase in PaCO_2_. Furthermore, it is assumed that a reduced interaction between blood and foreign material, i.e., a preferably small device, may potentially support biocompatibility [44]. The streamlined design of Prismalung+ might require less anticoagulation, which entails a lower risk of bleeding complications in patients, as there is less potential for pooling and low flows of blood within the device. This hypothesis needs to be investigated in future studies.

An advantage of lower flow ECCO_2_R devices is that smaller bore catheters can be used. A second advantage is that they may be integrated with other organ support strategies familiar to critical care physicians and nurses, such as continuous renal replacement therapy (CRRT), making these approaches much more feasible in the busy critical care environment. The potential to integrate ECCO_2_R into continuous renal replacement circuits may improve the risk/benefit ratio for hypercapnic patients with acute kidney injury (AKI) [45]. If effective, such devices could also be used in patients that do not have AKI, given the familiarity of the critical care team with this equipment. A feasibility study demonstrated that the use of a low-flow ECCO_2_R device managed with an RRT platform easily and safely enabled very-low-tidal-volume ventilation with moderate increase in PaCO_2_ in patients with mild-to-moderate ARDS [43].

### Comparison of CO_2_ removal by different devices

In this study, the new PrismaLung+ device performed similarly to the A.L.ONE device, with both devices demonstrating CO_2_ removal rates ~ 50% greater than the PrismaLung device. While the increase in CO_2_ removal observed with PrismaLung+ compared with PrismaLung can, at least in part, be explained by an increase in membrane surface area from 0.35 m^2^ to 0.8 m^2^, the similarities observed for PrismaLung+ and A.L.ONE occurred despite an increase in surface area, suggesting a more complex explanation. Recent data from Karagiannidis and colleagues suggest that the capability of different ECCO_2_R devices to eliminate CO_2_ is dependent upon a dynamic interplay within the device between the surface area available for gas exchange and the blood flow rate [44]. Devices with gas exchange membrane surface areas ranging from 0.35 m^2^ (e.g., PrismaLung) up to 1.3 m^2^ (e.g., A.L.ONE) are currently used in clinical practice [19, 22, 26, 46–48]. Furthermore, recent in vitro and in silico studies suggest that CO_2_ removal rate can increase with increasing blood flow rate [49, 50], in line with the observations we report here. Our study also confirms the findings from Karagiannidis and colleagues [44], as both surface area and blood flow rates govern the rate of CO_2_ removal; however, an increase in the surface area above a certain threshold has limited impact on CO_2_ removal when low blood flows are applied, as is the case with the A.L.ONE device. Larger membrane surface areas are thought to result in greater levels of CO_2_ removal at higher blood flow rates, with a smaller pressure drop across the gas exchanger [44]. In our in vitro study, pressure drop values across the three devices were relatively low, with levels of up to 25 mmHg with PrismaLung+ (surface area 0.8 m^2^) at a blood flow rate of 450 mL/min. Some challenges do exist for devices with larger membrane surface areas and those that require larger priming volumes, as they may have increased thrombotic potential due to increased interaction with an artificial surface [44]. A lower blood flow rate combined with a larger surface area may lead to more clotting events due to the increased time blood spends passing through the membrane [43, 51]. Furthermore, in the clinic, larger priming volumes can negatively affect exposure time and the hemolysis index [51], potentially resulting in increased blood loss due to clotting events and the device having to be replaced. We did not observe clotting events; however, this was not a focus of the study and would require investigation in the clinic.

These findings highlight the potential for lower flow devices with higher surface areas to remove CO_2_ from blood. The data also demonstrate the restrictions of conventional diffusive CO_2_ removal determined by blood flow rates. To further enhance CO_2_ removal at low blood flow rates novel systems, such as approaches involving acidification of bloo d[52] or bicarbonate dialysi s[53], need to be investigated.

### Study limitations and considerations

We used the infusion analysis rather than the more commonly accepted blood gas analysis method to determine the CO_2_ removal rates. This was done because of the limitations of the blood gas analysis method, because test conditions utilizing lower blood flow produced CO_2_ results below the reportable range of the analyzer. The blood gas analysis method was used to determine CO_2_ removal rates when test results where within the reportable range of the blood gas analyzer. The results obtained with the blood gas analysis method demonstrated the validity of the infusion method. The comparison of the valid data obtained by blood gas analysis versus the infusion method by slope analysis indicated that these two data sets are comparable (Fig. 5). It is important to note that the comparative data indicated that a small amount of gas loss from the test setup was likely, as for example, if the reservoir and/or tubing are not fully gas-tight. This is demonstrated by the data being slightly off the line of identity, the offset of the mean, as shown in the Bland–Altman diagram (4.2 mL/min) and the paired *t* test.

The general suitability of the blood gas analyzer, with respect to its intended use, was verified through the measurement principles of the analyzer [32, 33], to ensure that the ABL90 device used here could measure bovine blood parameters. In addition, the ABL devices from Radiometer have been routinely used to perform experiments on blood from different species [54–57].

We used a flow rate that would be achievable by a monitor that the PrismaLung+ device is intended to be used on in the clinic, namely 200 to 450 mL/min, allowing us to characterize the device in conditions comparable to mild-to-moderate hypercapnia where the device would be used. The A.L.ONE device is designed to run at higher blood flow rates than the flow rates used in this study; therefore, conditions perhaps did not favor the CO_2_ removal rate of the device, despite the high surface area in comparison with PrismaLung+ [31].

Further methodological limitations to consider include the use of bovine blood for experimentation, as it is easier to obtain than human blood and well-accepted for use in in vitro studies. It should be noted that as the blood was obtained from healthy animals, levels of blood components will be different from those for ICU patients; however, levels were consistent across experiments. Furthermore, a high dose (5 U/mL) of heparin was used that is higher than that routinely used in the clinic; this dose was selected to ensure no clotting occurred during transportation from the slaughterhouse and during the in vitro experiment and is not expected to impact CO_2_ removal. Given the in vitro nature of the data, caution should be exercised when translating these data to the clinical setting, and further studies are needed to explore coagulation in the clinic.

Here, and in similar studies, the CO_2_ removal rates are stated in units of mL/min, with dependency upon pressure and chosen reference temperature. To remove the dependency upon these parameters, units of mmol/min would be more appropriate and comparable when reporting CO_2_ removal rates. Despite this, the units of mmol/min are not standard and are not used in the clinical setting_._

ECCO_2_R uses similar gas-exchange principles as ECMO, but the main goal is to remove CO_2_ in those with sufficient oxygenation and at lower blood flow rates than ECMO [58]. Oxygenation and O_2_ transfer rates were not a focus in this study. In the clinical setting, venous sO_2_ levels are expected to be around 70% and therefore the testing of fully oxygen-saturated blood in this setting may slightly underestimate CO_2_ removal performance. This will require further experimental confirmation.

## Conclusions

In summary, at the flow rates tested, PrismaLung+ performed more effectively than PrismaLung for CO_2_ removal, with comparable performance to A.L.ONE, despite the smaller surface area. The PrismaLung+ may be an effective device for reducing CO_2_ levels, and further testing in the clinical setting is warranted.

## Supplementary information

**Additional file 1: Figure S1.** Cross section of the PrismaLung+ device

**Additional file 2: Figure S2.** CO_2_ removal rates for the PrismaLung+ and A.L.ONE devices. Assessed at 37 °C, *Q*_B_ 600 mL, and a p_in_CO_2_ of 45 mmHg. p_in_CO_2_, partial pressure of carbon dioxide at the inlet; *Q*_B_, blood flow rate. Data are plotted as mean values ± SD. *p* > 0.05 PrismaLung+ vs. A.L.ONE, not significantly different. Results are the mean of 4 tested devices

## Data Availability

The datasets used and/or analyzed during the current study are available from the corresponding author on reasonable request.

## Appendix

**Table 3:** Test conditions at the inlet for 9 different test settings

		PrismaLung+					PrismaLung					A.L.ONE				
Set *Q*_B_, mL/min	Set p_in_CO_2_, mmHg	ctHb, g/dL	pH	p_in_CO_2_, mmHg	pO_2_, mmHg	FMetHb, %	ctHb, g/dL	pH	p_in_CO_2_, mmHg	pO_2_, mmHg	FMetHb, %	ctHb, g/dL	pH	p_in_CO_2_, mmHg	pO_2_, mmHg	FMetHb, %
200	45	11.9 (0.2)	7.4 (0.0)	45.4 (2.2)	169.3 (11.6)	2.5 (0.3)	11.7 (0.2)	7.4 (0.0)	45.3 (3.2)	162.7 (7.2)	2.5 (0.3)	11.9 (0.3)	7.4 (0.0)	45.6 (1.1)	170.0 (4.3)	2.7 (0.4)
300	45	12.0 (0.2)	7.3 (0.0)	45.4 (1.9)	166.8 (1.1)	2.6 (0.2)	11.8 (0.2)	7.4 (0.0)	45.7 (1.5)	163.1 (3.0)	2.6 (0.3)	11.9 (0.3)	7.4 (0.0)	44.5 (1.3)	165.8 (2.9)	2.8 (0.2)
450	45	12.0 (0.3)	7.3 (0.0)	45.7 (1.7)	164.0 (0.8)	2.7 (0.1)	11.8 (0.3)	7.4 (0.0)	45.3 (0.9)	160.1 (2.6)	2.6 (0.1)	11.9 (0.3)	7.4 (0.0)	44.7 (1.1)	161.6 (2.6)	2.8 (0.3)
200	60	11.9 (0.2)	7.3 (0.0)	58.7 (2.7)	174.4 (2.5)	2.6 (0.2)	11.8 (0.3)	7.3 (0.0)	61.0 (1.5)	168.9 (5.7)	2.7 (0.1)	11.9 (0.3)	7.3 (0.0)	59.6 (1.3)	172.9 (5.3)	2.7 (0.3)
300	60	12.0 (0.3)	7.3 (0.0)	59.9 (2.2)	173.1 (1.5)	2.7 (0.1)	11.9 (0.2)	7.3 (0.0)	57.7 (0.7)	166.4 (3.7)	2.7 (0.1)	11.9 (0.3)	7.3 (0.0)	59.8 (2.0)	171.5 (3.7)	2.9 (0.2)
450	60	12.0 (0.2)	7.3 (0.0)	61.3 (1.8)	170.9 (1.3)	2.9 (0.2)	11.9 (0.2)	7.3 (0.0)	60.9 (1.6)	164.2 (2.6)	2.8 (0.1)	11.9 (0.3)	7.3 (0.0)	59.3 (2.0)	168.1 (3.1)	2.8 (0.2)
200	80	12.0 (0.3)	7.2 (0.0)	82.5 (2.9)	181.7 (5.3)	2.8 (0.2)	11.8 (0.3)	7.2 (0.0)	80.7 (3.5)	172.7 (10.5)	2.8 (0.2)	12.0 (0.3)	7.2 (0.0)	81.0 (1.8)	182.7 (4.5)	2.9 (0.2)
300	80	12.0 (0.2)	7.2 (0.0)	82.7 (2.9)	179.9 (2.8)	2.9 (0.2)	11.8 (0.3)	7.2 (0.0)	80.6 (1.7)	173.8 (3.5)	2.9 (0.2)	11.9 (0.3)	7.2 (0.0)	79.4 (2.8)	179.9 (2.4)	3.0 (0.1)
450	80	12.0 (0.2)	7.2 (0.0)	79.4 (2.1)	177.4 (2.3)	3.0 (0.2)	11.8 (0.3)	7.2 (0.0)	82.0 (2.2)	169.5 (2.1)	2.9 (0.1)	11.9 (0.3)	7.2 (0.0)	80.7 (2.4)	176.3 (2.7)	3.0 (0.1)

Values are mean ± SD. Means include measurements from 5 tested devices per condition

*ctHb* total hemoglobin concentration, *FMetHb* fraction of methemoglobin, *p*_*in*_*CO*_*2*_ partial pressure of carbon dioxide at the inlet, *pO*_*2*_ partial pressure of oxygen, *Q*_*B*_ blood flow rate

**Table 4:** CO_2_ removal rates using the infusion method at 37 °C for PrismaLung+, PrismaLung, and A.L.ONE devices

		Mean normalized JCO_2_ removal rate, mL/min (SD)			*p* value	
Set *Q*_B_, mL/min	Set p_in_CO_2_, mmHg	PrismaLung+	PrismaLung	A.L.ONE	PrismaLung+ vs. PrismaLung	PrismaLung+ vs. A.L.ONE
200	45	54 (3.8)	35 (2.5)	54 (0.7)	< 0.001	0.62
300	45	73 (4.0)	44 (2.5)	72 (1.9)	< 0.001	0.59
450	45	93 (4.0)	54 (3.2)	92 (2.7)	< 0.001	0.63
200	60	65 (5.0)	43 (1.9)	64 (1.4)	< 0.001	0.70
300	60	86 (5.7)	55 (3.0)	85 (2.8)	< 0.001	0.66
450	60	111 (7.8)	66 (3.4)	110 (4.3)	< 0.001	0.76
200	80	75 (6.1)	52 (2.4)	74 (2.7)	< 0.001	0.84
300	80	101 (5.8)	66 (2.8)	100 (3.0)	< 0.001	0.80
450	80	133 (7.5)	81 (3.2)	131 (4.7)	< 0.001	0.55

*JCO*_*2*_ normalized CO_2_ removal rate, *p*_*in*_*CO*_*2*_ partial pressure of carbon dioxide at the inlet, *Q*_*B*_ blood flow rate, *SD* standard deviation

## Abbreviations

AKI: acute kidney injury
ARDS: acute respiratory distress syndrome
BGA: blood gas analysis
COPD: chronic obstructive pulmonary disease
ECCO_2_R: extracorporeal CO_2_ removal
ECMO: extracorporeal membrane oxygenation
IMV: invasive mechanical ventilation
IUPAC: International Union of Pure and Applied Chemistry
LPV: lung-protective ventilation
MV: mechanical ventilation
NIV: non-invasive positive pressure ventilation
PBW: per body weight
pCO_2_: partial pressure of carbon dioxide (CO_2_)
p_in_CO_2_: partial pressure of carbon dioxide (CO_2_) at the inlet
PEEP: positive end-expiratory pressure
*Q*_B_: blood flow rate
RRT: renal replacement therapy
SD: standard deviation
STP: standard temperature and pressure
VILI: ventilator-induced lung injury
*V*_T_: tidal volume
