# Supplementary figures and images for: In vitro characterization of PrismaLung+: a novel ECCO2R device
Source: Intensive Care Med Exp. 2020 May 13;8:14. doi: 10.1186/s40635-020-00301-7 (PMC7221037; doi:10.1186/s40635-020-00301-7)

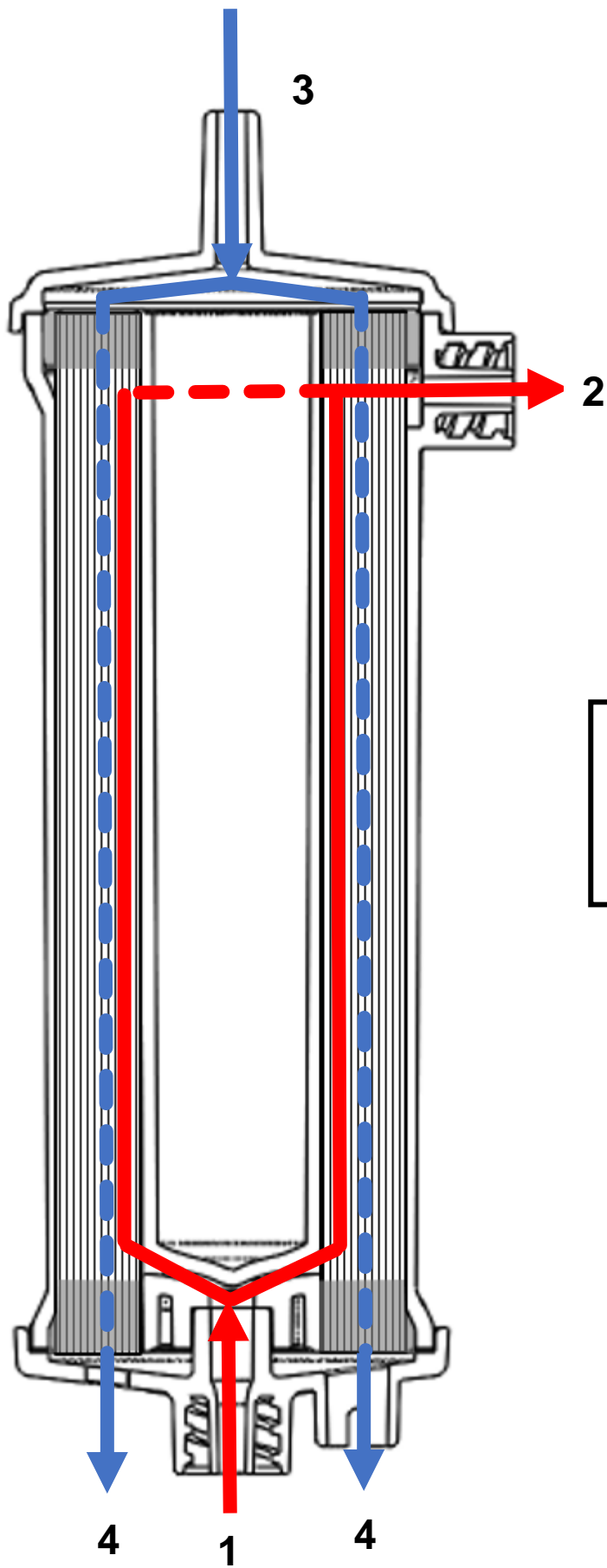

- 1 Blood inlet
- 2 Blood outlet
- 3 Gas inlet
- 4 Gas outlet

Supplement: Supplementary file 1 — Additional file 1: Figure S1. Cross section of the PrismaLung+ device [file 40635_2020_301_MOESM1_ESM.pdf]

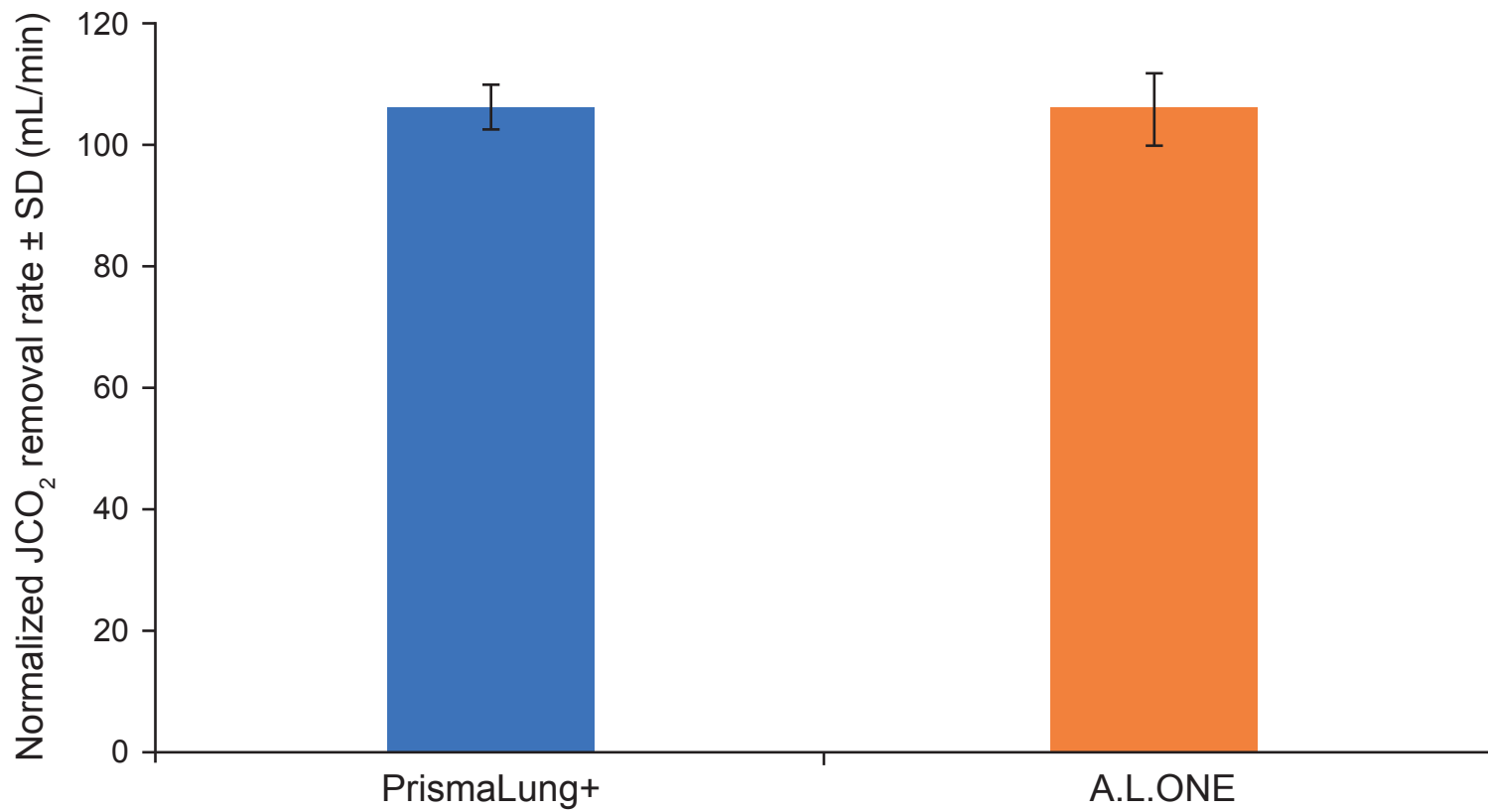

Supplement: Supplementary file 2 — Additional file 2: Figure S2. CO2 removal rates for the PrismaLung+ and A.L.ONE devices. Assessed at 37 °C, QB 600 mL, and a pinCO2 of 45 mmHg. pinCO2, partial pressure of carbon dioxide at the inlet; QB, blood flow rate. Data are plotted as mean values ± SD. p > 0.05 PrismaLung+ vs. A.L.ONE, not significantly different. Results are the mean of 4 tested devices [file 40635_2020_301_MOESM2_ESM.pdf]
